# Protection of Ischemic Postconditioning against Neuronal Apoptosis Induced by Transient Focal Ischemia Is Associated with Attenuation of NF-κB/p65 Activation

**DOI:** 10.1371/journal.pone.0096734

**Published:** 2014-05-06

**Authors:** Jianmin Liang, Yongxin Luan, Bin Lu, Hongbo Zhang, Yi-nan Luo, Pengfei Ge

**Affiliations:** 1 Department of Pediatrics, First hospital of Jilin University, Changchun, China; 2 Department of Neurosurgery, First hospital of Jilin University, Changchun, China; 3 Neuroscience Research Center, First hospital of Jilin University, Changchun, China; National University of Singapore, Singapore

## Abstract

**Background and Purpose:**

Accumulating evidences have demonstrated that nuclear factor κB/p65 plays a protective role in the protection of ischemic preconditioning and detrimental role in lethal ischemia-induced programmed cell death including apoptosis and autophagic death. However, its role in the protection of ischemic postconditioning is still unclear.

**Methods:**

Rat MCAO model was used to produce transient focal ischemia. The procedure of ischemic postconditioning consisted of three cycles of 30 seconds reperfusion/reocclusion of MCA. The volume of cerebral infarction was measured by TTC staining and neuronal apoptosis was detected by TUNEL staining. Western blotting was used to analyze the changes in protein levels of Caspase-3, NF-κB/p65, phosphor- NF-κB/p65, IκBα, phosphor- IκBα, Noxa, Bim and Bax between rats treated with and without ischemic postconditioning. Laser scanning confocal microscopy was used to examine the distribution of NF-κB/p65 and Noxa.

**Results:**

Ischemic postconditioning made transient focal ischemia-induced infarct volume decrease obviously from 38.6%±5.8% to 23.5%±4.3%, and apoptosis rate reduce significantly from 46.5%±6.2 to 29.6%±5.3% at reperfusion 24 h following 2 h focal cerebral ischemia. Western blotting analysis showed that ischemic postconditioning suppressed markedly the reduction of NF-κB/p65 in cytoplasm, but elevated its content in nucleus either at reperfusion 6 h or 24 h. Moreover, the decrease of IκBα and the increase of phosphorylated IκBα and phosphorylated NF-κB/p65 at indicated reperfusion time were reversed by ischemic postconditioning. Correspondingly, proapoptotic proteins Caspase-3, cleaved Caspase-3, Noxa, Bim and Bax were all mitigated significantly by ischemic postconditioning. Confocal microscopy revealed that ischemic postconditioning not only attenuated ischemia-induced translocation of NF-κB/p65 from neuronal cytoplasm to nucleus, but also inhibited the abnormal expression of proapoptotic protein Noxa within neurons.

**Conclusions:**

We demonstrated in this study that the protection of ischemic postconditioning on neuronal apoptosis caused by transient focal ischemia is associated with attenuation of the activation of NF-κB/p65 in neurons.

## Introduction

Ischemic stroke due to lack of cerebral blood supply is one of the most common causes leading to death or disability in adults worldwide [1]. Either animal study or clinical finding has revealed that reperfusion following ischemia results in brain damage [2], [3]. Since it was found that the activation of nuclear factor κB (NF-κB) induced by transient ischemia is prior to DNA fragmentation [4], accumulating evidences have demonstrated that NF-κB plays an important role in regulating transient ischemia-induced neuronal death [5], [6]. NF-κB is a nuclear transcription factor comprising five different proteins including p50, RelA/p65, c-Rel, RelB and p52, of which RelA/p65 and p50 have been proved to be responsible for the detrimental effect of NF-κB on neuronal injury in cerebral ischemia [7]. Schneider et al found that transient ischemia-induced brain damage and neuronal death reduced in NF-κB/p50 deficient mice when compared with that in wild type mice [8]. By contrast, inhibition of NF-κB/65 is found to underlie the protective mechanism of many compounds against brain damage caused by transient ischemia [9], [10]. In resting cells, NF-κB is normally sequestered in the cytoplasm by binding to its inhibitory IκB proteins. Under stress conditions such as ischemia and hypoxia, IκB is phosphorylated by its kinase (IKK), which leads to its degradation and disruption of the NF-κB/IκB complex. The activated NF-κB translocates subsequently to nucleus and binds to the κB promoter region of target genes [7]. Within neurons, NF-κB activation up-regulates the expression of pro-apoptotic factors such as Noxa and Bim [11]. By contrast, the activated NF-κB in glial cells could induce the production of neuro-toxic cytokines such as IL-1β, TNF-α and IL-6, which makes secondary injury to neurons [12]. Therefore, regulating the activation of NF-κB has become the target to prevent neuronal injury caused by transient cerebral ischemia.

Ischemic postconditioning, as a procedure consisting of series of rapid intermittent interruptions of blood flow in the early phase of reperfusion, is effective in protecting cerebral damage caused by ischemia/reperfusion [13]. Both animal studies and clinical investigation showed that ischemic postconditioning has protective effects on transient ischemia-induced injury. Wang et al and Ren et al reported respectively that ischemic postconditioning protected rat cerebral injury caused by either transient global or focal ischemia [14], [15]. Loukogeorgakis et al observed that ischemic postconditioning attenuated endothelial injury secondary to transient ischemia in human brachial artery [16]. Because ROS (reactive oxygen species) is an important trigger of the activation of NF-κB [17] and the protective effect of ischemic postconditioning on ischemic brain injury is correlated with inhibition of oxidative stress [18], [19], we hypothesize that the neuro-protection produced by ischemic postconditioning on transient ischemia-induced brain damage and neuronal apoptosis might be via regulating the activation of NF-κB. Therefore, in this study, we used rat model of transient focal ischemia to investigate the effect of ischemic postconditioning on the activation of NF-κB.

## Materials and Methods

### Animals

Adult male Wistar rats (weighing 280–300 g; 7 to 8 weeks of age) supplied by Experimental Animal Center, Jilin University, Changchun, China, were housed in a temperature-controlled room (22–25°C) on a 12-h light/dark cycle with free access to food and water. All animal procedures were approved by the ethical committee for animal experiments, Jilin University. All possible measures were taken to reduce animal suffering and numbers of animals in this study.

### Surgical procedure

Brain ischemia was produced by using the middle cerebral artery occlusion (MCAO) model in rats as described before [18]. Following overnight fast of the rats, anesthesia was induced with intra-peritoneal administration of chloral hydrate (300 mg/kg). A rectal probe was inserted and core temperature was maintained at 37±0.5°C with a heating pad and lamp. A surgical incision was made to expose the right common carotid artery (CCA), internal carotid artery, and external carotid artery. The external carotid artery was ligated proximal to the origin of any branches, such as the occipital artery. The proximal CCA then was ligated and temporarily closed proximal to the carotid bifurcation by a microvascular clip. A small incision was made in the CCA. The occlusion filament was inserted into the internal carotid artery through the CCA 19 to 21 mm distal from the bifurcation to occlude the origin of the MCA. The filament was prepared of monofilament fishing line and covered with a distal cylinder of silicon rubber (diameter 0.31 to 0.32 mm). After the MCAO was performed, animals were allowed to awaken and resume spontaneous breathing. Two hours after induction of ischemia, the filament was withdrawn. After surgery, animals were then placed into a cage to recover from anesthesia at room temperature and were allowed food and drink.

### Ischemic postconditioning protocol

At the start of the study, the rats were assigned randomly into a sham-operated group, an ischemia group and an ischemic postconditioning group. The rats in the ischemia group and ischemic postconditioning group were subjected to 2 h of focal cerebral ischemia as described above. The rats in ischemia group were subjected to 2 h of ischemia only, without any further interruption of reperfusion. Ischemic postconditioning rats were subjected to three cycles of 30 seconds/30 seconds reperfusion/reocclusion after 2 h of ischemia. In sham group, rats were subjected to the same procedures except for occlusion of the MCA.

### Evaluation of neurological functional score

Neurological functional scores were evaluated at 24 hours post reperfusion by randomly choosing six rats from each group by staffs blinded to these groups. The test consists of two aspects of neurological function as has been previously described [20]: (1) the postural reflex test to examine upper body posture while the animal is suspended by the tail; (2) the forelimb placing test to examine sensorimotor integration in forelimb placing responses to visual, tactile, and proprioceptive stimuli. Neurological function was graded on a scale of 0 to 12 (normal score, 0; maximal score, 12).

### Measurement of infarct size

At 24 h after reperfusion, seven rats from each group were chosen randomly by staffs who were blinded to these rats, killed by an overdose of pentobarbital i.p. and their brains were rapidly removed. Infarct sizes were measured by staining with 2, 3, 5-triphenyl-2H-tetrazolium chloride (TTC; Sigma-Aldrich, St. Louis, MO, USA). Brains were cut into 2-mm-thick coronal sections in a cutting block and 6 slices were stained with 1% TTC solution for 30 min at 37°C followed by overnight immersion in 4% paraformaldehyde. The percentage of brain infarct was measured by normalizing to the entire brain tissue from the animals, as described previously [18].

### Brain tissue fixation

After rat was anesthetized, the thorax was opened and the heart was disclosed. Herpin (0.1 mL, 300 IU/kg) was injected into the left ventricle before the catheter was inserted into the main artery via left atrium. Then, PBS was perfused into the vascular system at 4°C for 3 min, and PBS with 4% paraformaldehyde was perfused at 4°C for another 3 min. Subsequently, the brain tissue was taken out and put into PBS fixation solution containing 4% paraformaldehyde at 4°C. Twelve hours later, 20- µm and 50- µm coronal brain slices were cut by vibrotome and the brain slices in similarity were selected for TUNEL staining and immunohistochemistry labeling.

### TUNEL staining

TUNEL staining was performed at reperfusion 24 h by using In Situ Cell Death Detection Kit (Roche, Mannheim, Germany) according to the manufacturer's protocol. Brain slices were post-fixed 5 min in ethanol acetic acid (2∶1) and rinsed. Then, the sections were incubated in proteinase K (20 µg/ml) for 15 min, followed by 10 min quenching in 3% hydrogen peroxide at room temperature. After three 10 min washes in PBS, the slices were incubated with TUNEL reaction mixture for 1 h at 37°C. Sections were washed in PBS three times for 10 min each and color development was performed in the dark with DAB (3,3′-diaminobenzidine). Hematoxylin was used for counter-staining, and the slices were finally mounted onto gelatin-coated slides and dried in dark room. TUNEL-positive apoptotic cells exhibited brown nuclear or cytoplasmic staining. In three fields of 0.04 mm^2^ at the dorsal, ventral, and middle border of the infarct region at the level of the anterior commissure, TUNEL-positive cells were counted by a blinded pathologist and expressed as percent of total cell count.

### Immunohistochemical analysis

The 50- µm brain slices were put into 24 wells plate with 500 µL citrate buffer (pH 6.0) and incubated in a 100°C water bath for 10 min. After cooling down, the slices were washed with the TBS solution containing 0.2% TX-100 for 10 min and blocked in the TBS solution containing 3% BSA and 0.2% TX-100 for 1 h at room temperature. Then they were incubated with mixtures of primary antibodies at 4°C overnight. For NF-κB/p65 staining, the primary antibody mixture included 1∶300 polyclonal NF-κB/p65(Cell signaling Technology, Danvers, MA, USA) and 1∶600 monoclonal anti-NeuN (Abcam, Cambridge, MA, USA) antibodies. For Noxa staining, the primary antibody mixture contained 1∶200 polyclonal Noxa (Santa Cruz Biotechnology) and 1∶600 monoclonal anti-NeuN (Abcam, Cambridge, MA, USA). After washed up with TBS solution containing 0.1%TX-100 for three times (10 min each) at room temperature, the antibody-labeled brain slices were incubated with 1% BSA containing 1∶500 Alexa Fluor 594 Donkey Anti-mouse IgG(Cell signaling Technology, Danvers, MA, USA)for 1 h at room temperature. After the brain slices were washed with TBS solution containing 0.1% TX-100 for three times (10 min), they were incubated with 1∶800 Alexa Fluor 488 Donkey Anti-rabbit IgG(Cell signaling Technology, Danvers, MA, USA). Again, the brain slices were washed with TBS solution containing 0.1% TX-100 for three times (10 min each), and then were mounted on slides and dried in dark room. Mounting medium was added on the slides prior to be covered with coverslips for observing by a laser scanning confocal microscope.

### Western blotting analysis

At reperfusion 6 h and 24 h, the brain tissues were isolated from ischemic penumbra cortices and homogenized in ice cold buffer (1.5 mmol/L Tris base-HCl pH 7.6, 1 mmol/L DTT, 0.25 mol/L sucrose, 1 mmol/L MgCl_2_, 1.25 µg/mL pepstatin A, 10 µg/mL leupeptin, 2.5 µg/mL aproptonin, 0.5 mmol/L PMSF, 2.5 mmol/L EDTA, 1 mmol/L EGTA, 0.1 mol/L Na_3_VO_4_, 50 mmol/L NaF, and 2 mmol/L sodium pyrophosphate). The homogenates were centrifuged (1000×g for 20 min at 4°C) and the protein concentration of the supernatants (containing cytoplasm) and pellets (containing nucleus) was measured. Western blot analysis was conducted with 10% sodium dodecyl sulfate-polyacrylamide gel electrophoresis (SDS-PAGE). Four samples (from four different rats) in every experimental group were used for statistical analysis. After electrophoresis, proteins were transferred onto PVDF membranes. The membranes were probed with the following primary antibodies: 1∶800 anti-Noxa (Santa Cruz Biotechnology, Santa Cruz, CA, USA); 1∶1000 anti-Caspase-3 (Abcam, Cambridge, MA,USA); 1∶1000 anti-NF-κB/p65, 1∶1000 anti-p-NF-κB/p65(Ser536), 1∶1000 anti-Bim, 1∶1000 anti-Bax, 1∶1000 anti-IκBα, 1∶1000 anti-p-IκBα, 1∶1000 anti-Lamin B1 and 1∶1000 anti-β-actin (Cell signaling Technology, Danvers, MA). Horseradish peroxidase-conjugated goat anti-rabbit secondary antibody was obtained from Cell Signaling Technology (Danvers, MA, USA). After being incubated with horseradish peroxidase-conjugated goat anti-rabbit IgG (1: 2000), blots were washed and immunoreactive proteins were visualized on a Kodak X-omat LS film (Eastman Kodak Company, New Haven, CT, USA) with an enhanced chemiluminescence. Densitometry was performed with Kodak ID image analyses software (Eastman Kodak Company).

### Statistical analysis

All data are expressed as mean ± SD and were analysed by SPSS statistical software, version 17.0 (SPSS Inc., Chicago, IL, USA) for Windows. One-way analysis of variance (ANOVA) was used for statistical comparisons between the different groups. *P*<0.05 was considered to be statistically significant.

## Results

### Ischemic postconditioning reduced cerebral infarction and neuronal apoptosis

**Figure 1:**
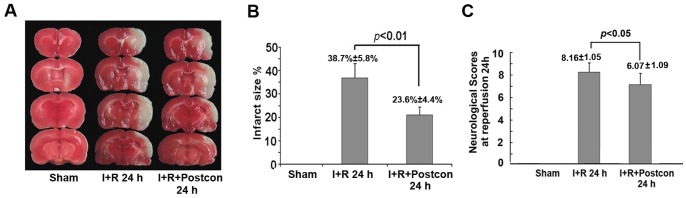
Measurement of cerebral infarction and evaluation of neurological function. A, representative images of TTC staining; B, statistics of infarct size. C, statistics of neurological scores.

In order to confirm the protection of the ischemic postconditioning procedure used in this study, we compared the infarct volume between ischemia group and ischemic postconditioning group. As shown in figure 1, the infarct volume at reperfusion 24 h was 38.7%±5.8% in ischemia group. By contrast, administration of ischemic postconditioning made the infarct volume decrease significantly to 23.6%±4.4% (*p*<0.01, versus ischemia group). This result indicated that 3 cycles of 30 seconds reperfusion/reocclusion is an effective ischemic postconditioning procedure to prevent brain injury. Moreover, neurological scores showed that ischemic postconditioning significantly mitigated the damaged neurological dysfunction caused by ischemia and reperfusion.

**Figure 2:**
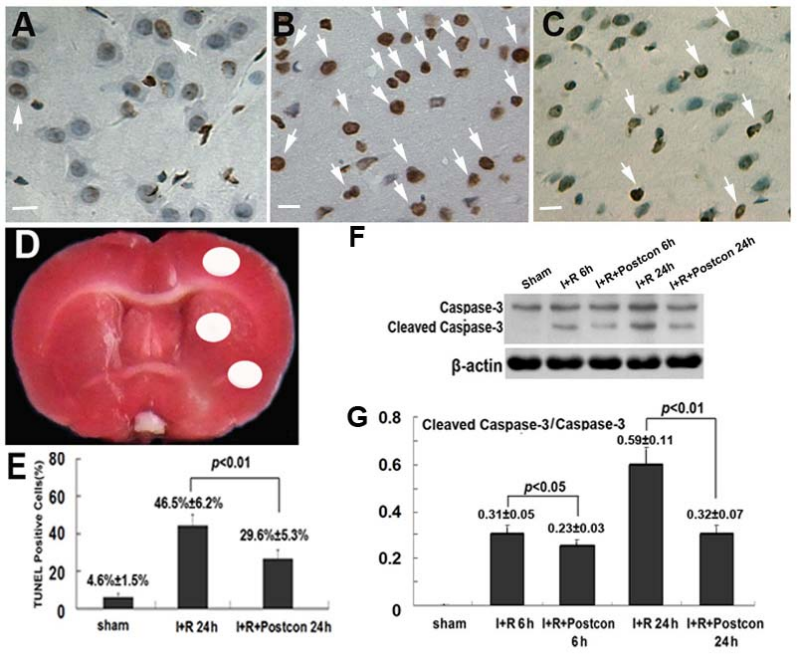
Detection of neuronal apoptosis. The representative images of TUNEL staining under microscope (x40) showed apoptotic cells exhibited brown nuclear or cytoplasmic staining (A, sham group; B, ischemia group; C, ischemic postconditioning group). D, brain region used for counting apoptotic neurons (White region). E, statistics of TUNEL positive cells. F, Western blotting of Caspase-3; G, statistics of the ratio of Cleaved Caspase-3 to Caspase-3. Scale bar: 20 µm.

Because previous reports showed that transient ischemia leaded to brain damage via apoptotic pathway [5], we compared the differences in the apoptosis rate at reperfusion 24 h between ischemia group and ischemic postconditioning group. As figure 2 showed, the apoptosis rate 4.6%±1.5% in the sham group was increased to 46.5%±6.2% by 2 h focal ischemia and 24 h reperfusion. However, ischemic postconditioning suppressed the elevated apoptosis rate to 29.6%±5.3% (*p*<0.01, versus ischemia group). For further examining the inhibitory effect of ischemic postconditioning on apoptosis, western blotting analysis was used to investigate the expressional changes in Caspase-3. It was found that ischemia/reperfusion induced cleavage of Caspase-3 was attenuated by administration of ischemic postconditioning (Figure 2 F and G). This result suggested the neuro-protection of ischemic postconditioning against ischemic brain damage is via inhibition of apoptosis.

### Ischemic postconditioning inhibited the translocation of NF-κBp/65 in neurons

**Figure 3:**
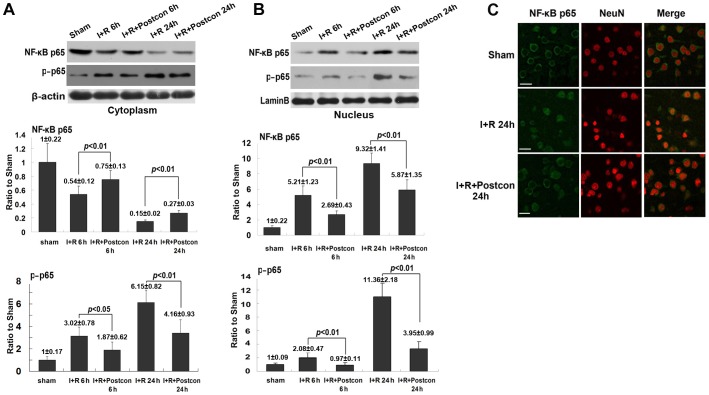
Analysis of the protein level of NF-κB/p65 and phosphor- NF-κB/p65 in cytoplasm and nucleus and observation of the distribution of NF-κB/p65 within neurons. A, representative western blotting image and statistics of NF-κB/p65 and phosphor- NF-κB/p65 in cytoplasm. B, representative western blotting image and statistics of NF-κB/p65 and phosphor- NF-κB/p65 in nucleus. C, representative confocal images of NF-κB/p65. This result showed that ischemic postconditioning reversed abnormal higher level of NF-κB/p65 and phosphor- NF-κB/p65, and the translocation of NF-κB/p65 from cytoplasm to nucleus caused by transient ischemia. Scale bar: 30 µm

When NF-κB/p65 is activated, it would translocate from cytoplasm to nucleus to induce apoptosis [11] and its transcriptional activity is also regulated by its phosphorylation by serine/threonine kinases[21]. Thus, we isolated cytoplasm fraction and nucleus fraction by using differential centrifugation and analyzed the protein level of NF-κB/p65 in each fraction by western blotting. As shown in figure 3 A and B, when compared with sham group, the protein level of NF-κB/p65 reduced in cytoplasm but increased correspondingly in nucleus either at reperfusion 6 h or 24 h. Moreover, its content in nucleus increased with the extension of reperfusion time from 6 h to 24 h. However, in the ischemic postconditioning group, the elevation of NF-κB/p65 in nucleus and its reduction in cytoplasm were both reversed significantly when compared with those in ischemia group at each indicated reperfusion time. Additionally, ischemic postconditioning significantly mitigated the phosphorylated level of NF-κB/p65 in both cytoplasm and nucleus induced by ischemia/reperfusion at either reperfusion 6 h or 24 h, indicating the transcriptional activity of NF-κB p65 was inhibited by ischemic postconditioning. In order to clarify whether ischemic postconditioning inhibited the translocation of neuronal NF-κB/p65 within neurons, we used laser scanning confocal microscopy in combination with neuron-specific probe NeuN to observe the distribution of NF-κB/p65. As shown in figure 3 C, the figures of nuclei were round and NF-κB/p65 was located in cytoplasm in sham group. At reperfusion 24 h following 2 h focal ischemia, nuclei shrank in some neurons and NF-κB/p65 distributed mainly in nuclei. By contrast, these changes in nuclei figures and distribution of NF-κB/p65 caused by ischemia and reperfusion were partly reversed by ischemic postconditioning. Therefore, our results indicated that ischemic postconditioning attenuated ischemia/reperfusion-induced translocation of NF-κB from cytosol to nucleus within cortex neurons.

### Ischemic postconditioning suppressed phosphorylation of IκBα

**Figure 4:**
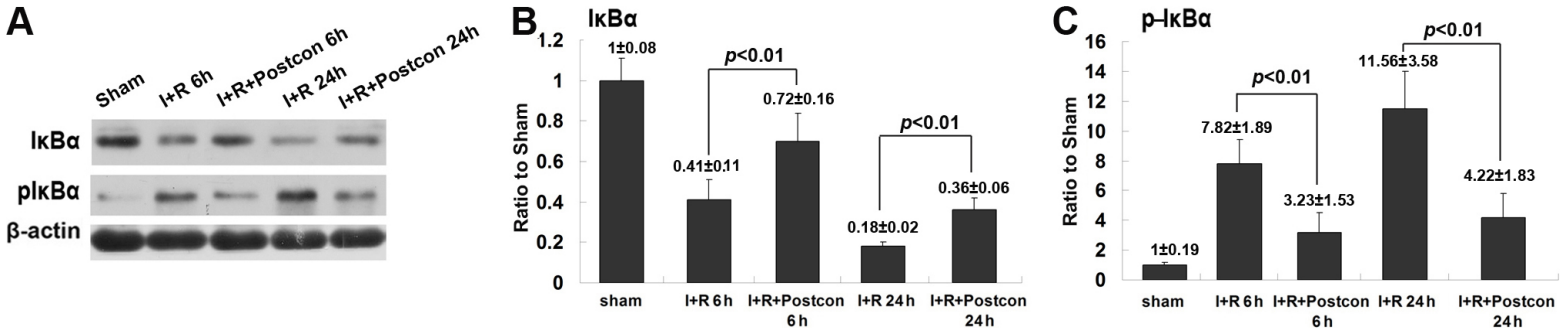
Analysis of the phosphorylation of IκBα. A representative image of western blotting; B, statistics of the protein level of IκBα; C, statistics of the protein level of phosphorylated IκBα. This result showed that ischemic postconditioning inhibited transient ischemia-induced phosphorylation of IκBα.

Despite there are several forms of IκB protein that have been identified, IκBα represents the predominant form in brain [22]. Thus, we examined the effects of ischemic postconditioning on the phosphorylation of IκB by using western blotting analysis. As shown in figure 4, compared with sham group, the level of phosphorylated IκBα increased significantly at reperfusion 6 h and 24 h, while the IκBα reduced markedly at each time point in ischemia group. Moreover, the reduction of IκBα was concomitant with the elevation of its phosphorylation when reperfusion time was extended from 6 h to 24 h. However, ischemic postconditioning suppressed markedly the phosphorylation of IκBα and maintained IκBα level in cytoplasm either at reperfusion 6 h or 24 h. This result indicated that the inhibitory effect of ischemic postconditioning on the activation of NF-κB/p65 is correlated with suppression of the phosphorylation of IκBα.

### Ischemic postconditioning suppressed the expression of proapoptotic proteins mediated by NF-κB

**Figure 5:**
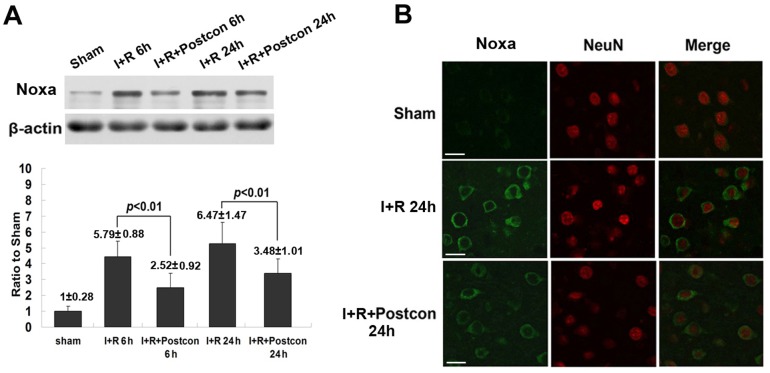
Analysis of the expressional level of Noxa and its changes within neurons. A, representative western blotting image of Noxa and statistical analysis; B, representative confocal images. This result showed that the up-regulated expression of Noxa in neurons was suppressed by ischemic postconditioning. Scale bar: 30 µm

**Figure 6:**
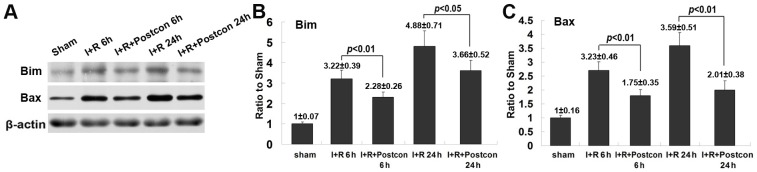
Analysis of the expressional level of Bim and Bax. A, representative western blotting images of Bim, Bax and cleaved caspase-3. B, statistics of the protein level of Bim; C, statistics of the protein level of Bax. This result showed that ischemic postconditioning mitigated the up-regulated expression of Bim and Bax.

The expressional level of proapoptotic proteins Noxa, Bim and Bax demonstrated to be regulated by activated NF-κB/p65 [7], [23] were investigated. As shown in figure 5 and figure 6, when compared with sham group, the expressional levels of pro-apoptotic proteins Noxa, Bim and Bax were all up-regulated markedly at both reperfusion 6 h and 24 h. Their expression at reperfusion 24 h was higher than those at reperfusion 6 h, respectively. However, the increased expressional levels of these three proapoptotic proteins were all suppressed at each indicated time by ischemic postconditioning. These results were consistent with the changes in NF-κB level in the rats treated with ischemic postconditioning, indicating that inhibition of the abnormal expression of these pro-apoptotic proteins was due to suppression of NF-κB activation by ischemic postconditioning. Further, we selected Noxa as a representative protein to investigate whether ischemic postconditioning mitigates its abnormal expression within neurons. As shown in figure 5, transient ischemia-induced elevation in the expression of Noxa was located in the cells labelled with neuron-specific probe NeuN. Obviously, administration of ischemic postconditioning mitigated the expression of Noxa within neurons. Thus, these results suggests mitigation of the expression of proapoptotic proteins Noxa, Bim and Bax is associated with the inhibitory effect of ischemic postconditioning on activation of NF-κB/p65.

## Discussion

In the present study, we demonstrated that ischemic postconditioning protected brain damage caused by transient focal ischemia through attenuating apoptotic neurons. Its inhibition of neuronal apoptosis was associated with suppressing ischemia/reperfusion-induced activation of NF-κB/p65 by mitigating over-phosphorylation of IκBα. Correspondingly, the expressional level of proapoptotic proteins Noxa, Bim and Bax mediated by NF-κB/p65 was reduced by ischemic postconditioning.

It has been found that NF-κB/p65 could be activated under various noxious stresses to mature brain, which include transient ischemia, subarachnoid hemorrhage, neurotrauma and epilepsy [5], [24]–[26]. Accumulating evidences suggest that activated NF-κB/p65 plays an important role in modulating cellular programmed death such as apoptosis and autophagy-like death. Nakai et al proved that activated NF-κB/p65 contributed to epilepsy-induced neuronal apoptosis in rat striatum [26]. Zeng et al reported that ischemia-induced activation of NF-κB/p65 aggravated myocardial injury through the activation of Beclin 1-mediated autophagy [27]. In the case of transient ischemia, it is found that activation of NF-κB/p65 is associated closely with neuronal damage [4], [5], and inhibition of the activation of NF-κB/p65 produces protection against neuronal apoptosis. Xu et al proved that inhibition of NF-κB/p65 signaling pathway by matrine contributed to its protective effect on neurons against cerebral focal ischemia [28]. Similarly, the protection of hypothermia and electrical acupuncture on brain damage was proved as well to be via modulating the activation of NF-κB/p65 [29], [30].Consistent with these reports, we not only demonstrated that transient ischemia induced activation of NF-κB/p65, but also found that ischemic postconditioning protects neuronal apoptosis caused by cerebral ischemia and reperfusion via inhibiting of NF-κB/p65 activation.

It was reported that NF-κB/p65 could be activated in both neurons and glial cells by transient ischemia, but their roles in ischemic brain damage is different. Zhang et al demonstrated by using transgenic mouse that ischemic damage is due to the activation of neuronal NF-κB/p65, not the glial NF-κB/p65 [5]. However, it is thought that transient ischemia-induced activation of NF-κB/p65 via toll like receptor 4 or 2 in glial cells would exert secondary injury to neurons by producing neuro-toxic cytokins such as TNFα and IL-1β [12]. In the present study, we demonstrated by using double immuno-fluorescence staining that ischemic postconditioning protects neuronal apoptosis via suppression of neuronal proapoptotic proteins. Although we did not investigate the effect of ischemic postconditioning on the activation of glial NF-κB, other researchers reported that ischemic postconditioning could inhibit the production of toxic cytokines TNFα and IL-1β induced by ischemia [31]. We thus speculate that the transient ischemia-induced activation of glial NF-κB/p65 might be inhibited by ischemic postconditioning.

Activated NF-κB/p65 promotes apoptosis in neurons via upregulating the expression of its downstream proapoptotic proteins, such as nitric oxide synthase II [32], Bax[23], Noxa and Bim[7]. Noxa and Bim are Bcl-2 family members, but only contain BH3 (Bcl-2 homology domain 3)-only domain. They both could activate Bax to release cytochrome c and other death signals from mitochondria, and prevent Bax from being blocked by anti-apoptotic Bcl-2 and Bcl-XL [33]. Noxa and Bim have been demonstrated to be associated with ischemic injury, because previous reports showed that the expression of Bim and Noxa were rup-regulated at the ischemic heart and brain [34], [11]. By contrast, suppression of the expression of Noxa by antisense oligonucleotides rescued hypoxia-induced cell death and decreased infarct volumes caused by focal cerebral ischemia [35]. In this study, we proved that ischemic postconditioning reduced the abnormal protein level of proapoptotic Noxa, Bim and Bax, which might underlie the inhibitive mechanism of ischemic postconditioning on neuronal apoptosis.

Despite it was reported that phosphorylation of NF-κB/p65 on Serine 536 defines an IκBα-independent NF-κB pathway[36], the activation of NF-κB/p65 is mainly regulated by its natural interior inhibitor IκB. Xu et al demonstrated by using recombinant adenoviral expression of dominant negative IκBα that over-expression of IκB rescued neuronal injury caused by cerebral ischemia [37]. The reduction of IκB during the course of cerebral ischemia and reperfusion is mainly due to its degradation by proteasome after it is phosphorylated by IκB kinase (IKK) [7]. However, it was found that NF-κB/p65 phosphorylation coincides with promotion of IκBα degradation [38]. In the present study, we demonstrated that ischemic postconditioning inhibited the ischemia/reperfusion-induced higher level of phosphorylated NF-κB/p65 in both cytoplasm and nucleus, which was consistent with the finding that ischemic postconditioning reversed the decreased level of IκBα caused by cerebral ischemia/reperfusion (figure 4).

Evidence showed that oxidative stress is an upstream event promoting IκB phosphorylation [10], [39]. Moreover, Shen et al reported antioxidant attenuated reperfusion injury after global brain ischemia via inhibiting NF-κB activity by mitigating the phosphorylation of IκB [40]. Similarly, Fischer et al proved that anti-oxidative treatment suppressed activation of NF-κB during myocardial ischemia-reperfusion in heart. [41]. On the basis of prior findings that ischemic postconditioning could attenuate the overproduction of ROS caused by transient focal ischemia and reverse the damaged activity in endogenous antioxidant enzyme [18], [42], we think that the inhibitory effect of ischemic postconditioning on the phosphorylation of IκB is also associated with its inhibition of oxidative stress.

The role of NF-κB/p65 during the course of cerebral ischemia and reperfusion is complex. In vitro and in vivo studies have shown that sublethal ischemia-induced activation of NF-κB/p65 contributed to the protection of ischemic preconditioning against subsequent lethal ischemic insult [43], [44]. Blondeau et al found that the protection of activated NF-κB/p65 against neuronal damage caused by following lethal stresses could be counteracted by diethyldithiocarbamate, an NF-κB/p65 specific inhibitor [44]. By contrast, other studies revealed that inhibiting the activation of NF-κB/p65 contributed to protection of tissue injury. Kin et al reported that the inhibition of myocardial apoptosis by postconditioning is associated with attenuation of NF-κB/p65 translocation [45]. Yin et al showed that preconditioning with mTOR inhibitor rapamycin rescued brain damage via attenuating the production of NF-κB/p65 caused by subsequent ischemia/reperfusion[46]. Although ischemic postconditioning and preconditioning might share common pathways to induce intrinsic protective mechanism such as modulation of ASIC1a, notch signaling and neuroinflammation, [47], [48], [31], our result showed that the effect of ischemic postconditioning on activation of NF-κB/p65 is inhibitive, which is opposite to the inducing effect produced by ischemic preconditioning.

It has been demonstrated that ischemic postconditioning protects transient ischemia-induced tissue damage in various organs, including heart, liver and intestine [13], [49], [50]. By now, the protective mechanism underlying ischemic postconditioning has been investigated widely and is found to be related to multiple factors including suppression of oxidative stress, maintaining mitochondrial function, inhibition of endoplasmic stress, attenuation of inflammation and mitigation of protein aggregation [51]–[54]. Different with previous reports showing that the inhibitory effect of ischemic postconditioning on cellular apoptosis is via down-regulating anti-apoptotic protein Bcl-2, up-regulating proapoptotic caspase-3, caspase-6 and caspase-9[55], and activation of ERK1/2 and Akt signaling pathway[56], [57], our result suggests that inhibition of neuronal apoptosis by ischemic postconditioning is related to attenuation of the activation of neuronal NF-κB/p65.

## Conclusion

Our study showed that ischemic postconditioning inhibited brain damage and neuronal apoptosis induced by transient ischemia. During the course of cerebral ischemia and reperfusion, the activation of NF-κB/p65 due to the phosphorylation of its inhibitor I-κBα and phosphorylation of NF-κB/p65 resulted in up-regulated expression of proapoptotic proteins Noxa, Bim and Bax. However, administration of ischemic postconditioning prior to the recovery of cerebral blood flow significantly inhibited these changes and rescued neuronal apoptosis. Therefore, our study indicates that ischemic postconditioning is an effective method that could protect neuronal apoptosis via attenuating the activation of NF-κB/p65 caused by focal cerebral ischemia and reperfusion.
